# Graphene Quantum Dots and Cu(I) Liquid Crystal for Advanced Electrochemical Detection of Doxorubicine in Aqueous Solutions

**DOI:** 10.3390/nano11112788

**Published:** 2021-10-21

**Authors:** Sorina Motoc Ilies, Bianca Schinteie, Aniela Pop, Sorina Negrea, Carmen Cretu, Elisabeta I. Szerb, Florica Manea

**Affiliations:** 1 “Coriolan Drăgulescu” Institute of Chemistry, Romanian Academy, 24 Mihai Viteazu Bvd., 300223 Timisoara, Romania; sorinailies@acad-icht.tm.edu.ro (S.M.I.); biancab@acad-icht.tm.edu.ro (B.S.); cretucarmen@acad-icht.tm.edu.ro (C.C.); 2 Department of Applied Chemistry and Engineering of Inorganic Compounds and Environment, Politehnica University of Timisoara, 2 Victoriei Square, 300006 Timisoara, Romania; aniela.pop@upt.ro; 3 National Institute of Research and Development for Industrial Ecology (INCD ECOIND), Timisoara Branch, 300431 Timisoara, Romania; negrea.sorina@yahoo.com; 4 Department of Environmental Engineering and Management, “Gheorghe Asachi” Technical University of Iasi, 700050 Iasi, Romania

**Keywords:** graphene quantum dots, liquid crystalline Cu(I) coordination complex, electrochemical detection, doxorubicine, C-based paste electrodes

## Abstract

Two paste electrodes based on graphene quantum dots and carbon nanotubes (GRQD/CNT) and one modified with a homoleptic liquid crystalline Cu(I) based coordination complex (Cu/GRQD/CNT) were obtained and morphostructurally and electrochemically characterized in comparison with simple CNT electrode (CNT) for doxorubicine (DOX) detection in aqueous solutions. GRQD/CNT showed the best electroanalytical performance by differential pulse voltammetry technique (DPV). Moreover, applying a preconcentration step prior to detection stage, the lowest limit of detection (1 ng/L) and the highest sensitivity (216,105 µA/mg·L^−1^) in comparison with reported literature data were obtained. Cu/GRQD/CNT showed good results using multiple pulse amperometry technique (MPA) and a favorable shifting of the potential detection to mitigate potential interferences. Both GRQD-based paste electrodes have a great potential for practical utility in DOX determination in water at trace concentration levels, using GRQD/CNT with DPV and in pharmaceuticals formulations using Cu/GRQD/CNT with MPA.

## 1. Introduction

In the last few decades, pharmaceuticals have been saving millions of lives and have emerged as a new class of environmental contaminant [1,2,3,4]. Since 1990, major efforts have been made to study the risks, occurrence, and fate of human pharmaceuticals (identified as pseudo-persistent compounds) which are constantly released in the environment [1,4]. These compounds can have both chronic and acute harmful effects on natural flora and fauna. According to Jureczko, 2020 [2], over six hundred pharmaceuticals were found in ground waters, surface waters (rivers, lakes, seas), and in soils.

Chemotherapy drugs are a specific group of pharmaceutical compounds used to treat cancer diseases. They are often called anticancer drugs and have been shown to have potent cytotoxic, genotoxic, mutagenic, carcinogenic, endocrine disruptor, and/or teratogenic effects in several organisms, since they have been mainly designed to disrupt or prevent cellular proliferation, usually by interfering in DNA synthesis [1,2,3,4,5,6].

Even if in the EU there are no legal standards for the presence of human cytostatic and cytostatic medical products in water, these compounds belong to emerging pollutants, and also, according to the EU Commission Decision 2000/532/EC251, they are included in European List of Hazardous Waste [2].

Nowadays, there is a lack of information about the occurrence and fate of these substances in the environment, although their consumption has increased in the last years and it is foreseen to further increase in the future due the increasing cases of cancers [4,6]. Their main sources are hospital effluents, household discharge and drug manufacturers. As these compounds are not removed during wastewater treatment with sufficient efficiency, they are found in the surface, ground and quite drinking water in concentrations at trace levels in general, up to 0.2 µg·L^−1^ [2]. For example, Doxorubicine (DOX), an anthracycline and anti-cancer chemotherapy drug, which is used in the treatment of various forms of sarcoma and cancer, including bladder cancer, breast cancer, leukemia, liver cancer, head and neck cancer, and lung cancer, has been detected in different matrices of water (hospital effluent: 1 × 10^−5^–1.5 × 10^−1^ mg·L^−1^, WWTP effluent < 4.3 × 10^−6^–4.20 × 10^−5^ mg·L^−1^; surface water <5.3 × 10^−6^ mg·L^−1^) [2]. It can be seen that the concentrations data are worrying, and it is evident that advanced analytical methods have to be developed. Due to the fact that the majority of analytical methods found in the literature are valid for biological matrix (blood, urine) [7,8,9], and not for water matrix, the detection of cytostatic, in special DOX, is difficult. Until now, several analytical techniques including high-performance liquid chromatography (HPLC), liquid chromatography-mass spectrometry (LS-MS), mass spectrometry methods have been developed to detect the trace levels of DOX in different matrices of water in concentrations up to 1 × 10^−7^ to 5 × 10^−5^ mg·L^−1^ DOX [2]. Although these methods have the advantages of sensitivity and accuracy, their high cost and complicated operations limit their applications.

Considering the high sensitivity, rapidity, accuracy, simplicity, low cost, ease of on-site determination, limit of detection at trace levels, the electrochemical methods have received considerable attention for the analysis of DOX [10,11,12,13,14,15].

The development of the electrochemical methods and sensing for the quantitative determination of the analytes address the two main issues regarding the electrochemical techniques and the electrode materials. In general, the electrochemical analysis is a simple, cost-effective method to determine the levels of electroactive species quantitatively and qualitatively in a solution. Advantages of the electroanalytical techniques over other detection methods such as chromatography, luminescence, and spectroscopy are their low cost, ease of use, accuracy, and reliability. Both advanced voltametric techniques (e.g., differential pulse voltammetry-DPV, square-wave voltammetry-SWV) besides the conventional (cyclic voltammetry-CV) and amperometric ones (chronoamperometry-CA and multiple-pulsed amperometry-MPA) require the optimization of the operating parameters, depending on the mechanism of the overall electrode process responsible for the detection response [16,17,18,19,20].

Carbon-based electrode material is very common and useful in electroanalysis but they are not always appropriate for detection at trace level concentrations due to slower electron mobility and implicit electrode process kinetics [21,22]. In addition, nanostructured carbon can be integrated within electrode composition to enhance the electroanalytical detection performance [17,18,19,20].

Graphene quantum dots (GRQD) as novel nanomaterials have received significant interest in the field of electroanalysis applications. They belong to zero-dimensional and sp^2^ hybridization, and are nanometer-sized fragments of graphene [23,24,25]. GRQD are superior in chemical inertness, simplicity of production, resistance to photobleaching, are environmentally friendly due to its non-toxic and biologically properties low cytotoxicity, and excellent biocompatibility in comparison to traditional semiconductor. Hence, these advantages make them applicable in sensors, bioimaging, optoelectronic devices, drug delivery, due to their high specific surface, electrical conductivity, and electrochemical mobility [24,25,26,27,28,29,30].

In order to better enhance the electroanalytical performance, chemically modified electrodes have been studied recently by our group, including metallic and metallic based Cu, Ag nanoparticles [17,18,19,20,31,32,33]. Very interesting behavior and application utility in sensitive sensing have been reported for homoleptic ionic Cu(I) coordination complex based on 2,2′-biquinoline ligand functionalized with long alkyl chains (Cu(I)_BF_4_) integrated within carbon nanofiber paste electrode [19].

In this study, the development of high performance electrochemical methods centered on GRQD based electrodes, GRQD integrated CNT paste electrode (GRQD/CNT) and homoleptic ionic Cu(I) coordination complex based on 2,2′-biquinoline ligand functionalized with long alkyl chains Cu/GRQD integrated CNT paste electrodes (Cu/GRQD/CNT paste electrode) for the quantitative determination of DOX in aqueous solutions. GRQD/CNT and CuGRQD/CNT paste electrodes were characterized morpho-structurally and electrochemically in comparison with simple CNT electrode. Advanced voltametric techniques, DPV and SWV, have been optimized to reach the highest sensitivity and the lowest limit of detection for DOX determination linked to the electrode compositions. To develop amperometric detection methods, CA and MPA techniques have been tested.

## 2. Materials and Methods

### 2.1. Materials

Graphene quantum dots (GRQD) were purchased from Sigma Aldrich (Saint Louis, MO, USA). Multiwall carbon nanotubes (CNT) synthesized by catalytic carbon vapor deposition (CCVD) were purchased from Nanocyl^TM^, Belgium. Cu(I)_BF_4_ complex was prepared as previously reported [34].

Standard stock solution of 1 g·L^−1^ Doxorubicin (DOX) was prepared daily from analytical grade Sigma Aldrich reagents using distillated double water. The supporting electrolyte for the characterization and application of electrode material in detection process was 0.1 M Na_2_SO_4_, and 0.1 M NaOH solutions, which were freshly prepared from Na_2_SO_4_, and NaOH, respectively, of analytical purity (Merck KGaA, Darmstadt, Germany) with double distillated water.

### 2.2. Preparation of the Working Paste Electrode

All working paste electrodes were obtained by simple mechanical mixing of certain amount of graphene quantum dots (GRQD), carbon nanotubes (CNT), homoleptic ionic Cu(I) coordination complex based on 2,2′-biquinoline ligand functionalized with long alkyl chains (Cu(I)_BF_4_) and paraffin oil, according to bellow presented Table 1.

### 2.3. Structural and Morphological Characterization

A Cary 630 FT-IR spectrophotometer was used to collect Fourier transform infrared spectroscopy (FTIR) spectra of GRQD/CNT, Cu/GRQD/CNT and CNT in paraffin oil paste at room temperature in the wavenumber range of 4000–400 cm^−1^ using transmission technique. A scanning electronic microscope (SEM, Inspect S PANalytical model) was used to characterize comparatively the morphological surfaces of GRQD/CNT, and Cu/GRQD/CNT paste electrode.

### 2.4. Electrochemical Experiments

Cyclic voltammetry (CV), differential-pulsed voltammetry (DPV), square-wave voltammetry (SWV), chronoamerometry (CA), and multiple-pulsed amperometry (MPA) were carried out using a computer controlled Autolab potentiostat/galvanostat PGSTAT 302N (EcoChemie, Utrecht, The Netherlands) controlled with Nova 2.4 software connected to a three-electrode cell consisting of a GRQD/CNT paste working electrode, and Cu/GRQD/CNT paste working electrode, a platinum counter electrode, and silver/silver chloride reference electrode (Ag/AgCl, KCl 3M).

The lowest limit of detection (LOD) was calculated using the equation: LOD = 3 SD/m and LQ = 10 SD/m, where SD is the standard deviation of three blanks and m is the slope of the analytical plots [35]. The reproducibility, (the relative standard deviation (RSD)) of the electrodes using the above-mentioned technique was evaluated for three replicates measurements of DOX detection.

## 3. Results and Discussion

### 3.1. Morphological Characterization

**Figure 1:**
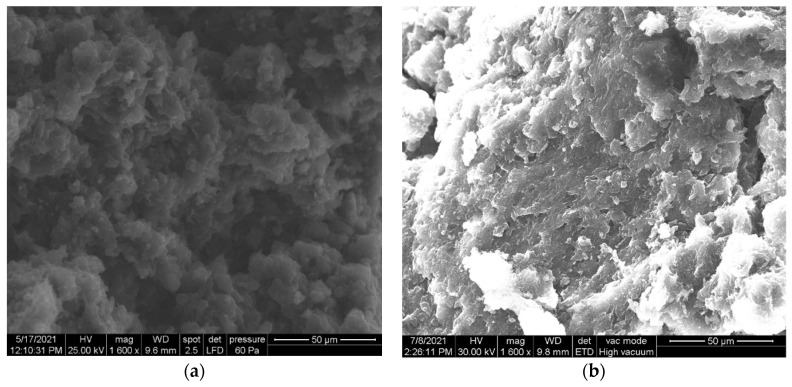
Scanning electron microscopy (SEM) images of: (**a**): GRQD/CNT; (**b**): Cu/GRQD/CNT.

Figure 1a,b show the SEM images of the GRQD/CNT and Cu/GRQD/CNT pastes. Even if the content of graphene quantum dots (GRQD) is lower in comparison with CNT, the presence of GRQD cover the interconnected tubular structure of CNT and a porous morphology is noticed (Figure 1a). The integration of a much larger amount of homoleptic ionic Cu(I) coordination complex based on 2,2′-biquinoline ligand functionalized with long alkyl chains (Cu(I)_BF_4_) within GRQD and CNT in the paste using paraffin oil is seen clearly by flakes-like lighter structure (Figure 1b).

The spectra of CNT and GRQD/CNT is dominated by the intense vibrations of aliphatic -CH_3_ and -CH_2_ groups of the paraffinic oil (See Figure S1a,b in Supplementary Materials): C–H stretching at 2857 and 2945 cm^−1^, C-H bending at 1457 and 1376 cm^−1^ and the rocking band at 721 cm^−1^, overlapping and strongly weakening the principal bands of the C-based materials (1580–1530 cm^−1^ (C-sp^2^) and 1200–1100 cm^−1^ (assigned to C=C bond from the CNT skeletal vibration mode) [36].

**Figure 2:**
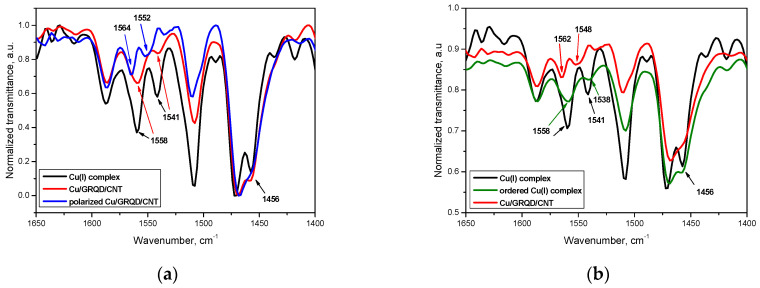
(**a**) 1650–1400 region of FT-IR spectra of Cu(I)_BF_4_, paste electrode Cu/GRQD/ CNT and polarized Cu/GRQD/ CNT; (**b**) 1650–1400 region of FT-IR spectra of complex Cu(I), thermally ordered complex Cu(I) and paste electrode Cu/GRQD/CNT.

The FT-IR spectra of the pristine complex Cu_BF4, paste electrode Cu/GRQD/CNT and polarized Cu/GRQD/CNT electrode are presented in Supplementary Materials (Figure S2). The spectra are overlapping on the whole IR region except for the 1610–1400 cm^−1^ region (Figure 2a), where the relative intensities corresponding to some bands assigned to C=C and/or C=N ring stretch vibration [37] are changing, suggesting a modification of the skeletal ring of the biquinoline ligand. This can be explained by a slight distortion of the complex structure when physically mixed with the C-based materials and paraffin oil, suggesting some kind of weak interactions between the complex and the functional groups on CNT and GRQD.

Cu(I)_BF_4_ complex is a liquid crystalline material, whose synthesis and characterization was reported previously [34]. When thermally ordered into mesophase from the pristine solid, similar distortions occur, caused by the closed-packing of the molecules into columnar structures, packing forces exerting a considerable influence on the molecular geometry of Cu(I) complexes bis-chelated with N^N-type ligands [38]. The modifications of the same bands in the FT-IR spectra of thermally ordered Cu(I)_BF_4_ with respect to the pristine solid showed in Figure 2b, strongly support the proposed distortions of the Cu(I) complex geometry in the paste electrode.

Moreover, after polarization the same region shows further alteration, with some small shift of the peaks maxima towards higher wavenumbers that can be caused by a further distortion and/or partial oxidation of copper center, with a partial recovery of the free ligand (Figure 2a).

### 3.2. Electrochemical Behavior of Carbon-Based Electrodes in the Presence of Supporting Electrolyte and 5 mg·L^−1^ DOX

**Figure 3:**
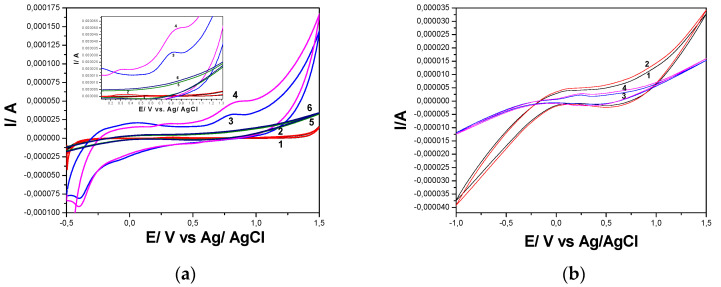
(**a**) Cyclic voltammograms recorded at: CNT paste electrode in 0.1 M Na_2_SO_4_ supporting electrolyte (curve 1), GRQD/CNT paste electrode in 0.1 M Na_2_SO_4_ supporting electrolyte (curve 3), and Cu/GRQD/CNT in 0.1 M Na_2_SO_4_ supporting electrolyte (curve 5) in the presence of 5 mg·L^−1^ DOX (curves 2, 4, and 6), potential scan rate: 0.05 V·s^−1^; potential range from −0.5 to +1.5 V/Ag/ AgCl. (**b**) Cyclic voltammograms recorded at Cu/GRQD/CNT paste electrode in: 0.1 M Na_2_SO_4_ supporting electrolyte (curve 1), and 0.1 M NaOH supporting electrolyte (curve 2), potential scan rate: 0.05 V·s^−1^; potential range from −1 to +1.5 V/Ag/ AgCl.

The electrochemical behaviors of the Cu(I)_BF_4_ liquid crystal/graphene quantum dots/carbon nanotubes (Cu/GRQD/CNT), graphene quantum dots/ carbon nanotubes (GRQD/CNT) paste electrodes in comparison with carbon nanotubes (CNT) paste electrodes was studied by CV in 0.1 M Na_2_SO_4_ supporting electrolyte and 5 mg·L^−1^ DOX (Figure 3a).

It can be easily seen that in the absence of DOX, the background current as a capacitive current attributed to the electric double layer is much higher for GRQD/CNT in comparison with CNT electrode. The introduction of a liquid crystalline coordination complex based on Cu(I) metal center, Cu(I)_BF_4,_ within the electrode composition by replacement of the corresponding GRQD significantly decreased its background in comparison with GRQD/CNT paste electrode, but it is still larger in comparison with CNT paste electrode. This behavior is attributed to the excellent properties of GRQD related the specific surface area, electrical conductivity, and electrocatalytic activity. It is noticed that several electrooxidation and electroreduction processes occurred on the GRQD, due to different interactions and physical connections between the active components from the paste electrode. The electrooxidation processes were noticed also on the Cu/GRQD/CNT and CNT paste electrodes at different potential values, higher for the CNT paste electrode in relation with the carbon structure and specific characteristics (0D-graphene quantum dots and 1D- CNT).

The electrochemical behavior of DOX onto the carbon-based electrodes showed the anodic peak attributed to DOX electrooxidation and the corresponding cathodic peak of the reduction process of DOX, which is noticed during the reverse scanning to cathodic range. It is observed that the DOX oxidation process started earlier onto the Cu/GRQD/CNT in comparison with GRQD/CNT and CNT paste electrode, which informs about an electrocatalytic effect towards to the DOX oxidation obtained probably due to the synergism between the Cu_BF_4_ and the C-based materials forming the paste electrode (see FT-IR results). On the other hand, the highest anodic peak height was achieved for GRQD/CNT paste electrode that is linked to the electrocatalytic effect and larger background current.

Considering in situ obtaining of copper oxides during scanning voltammetry running in alkaline medium [39], the comparative electrochemical behavior of Cu/GRQD/CNT paste electrode in 0.1 M Na_2_SO_4_ and 0.1 M NaOH supporting electrolytes was studied and the results are presented in Figure 3b. The anodic peak attributed to the first stage of copper oxidation is evidenced at the potential value of about +0.230 V vs. Ag/AgCl in alkaline medium in according with the literature data [39], which did not appear in 0.1 M Na_2_SO_4_ supporting electrolyte. Taking into account the better electrocatalytic activity of the copper oxides vs. copper ions, the alkaline medium is chosen for further applications of Cu/GRQD/CNT paste electrode for DOX detection.

To evidence the response of each electrode to DOX concentration increasing, CVs were recorded in 0.1 M Na_2_SO_4_ supporting electrolyte for GRQD/CNT and CNT paste electrodes and in 0.1 M NaOH supporting electrolyte for Cu/GRQD/CNT paste electrode.

**Figure 4:**
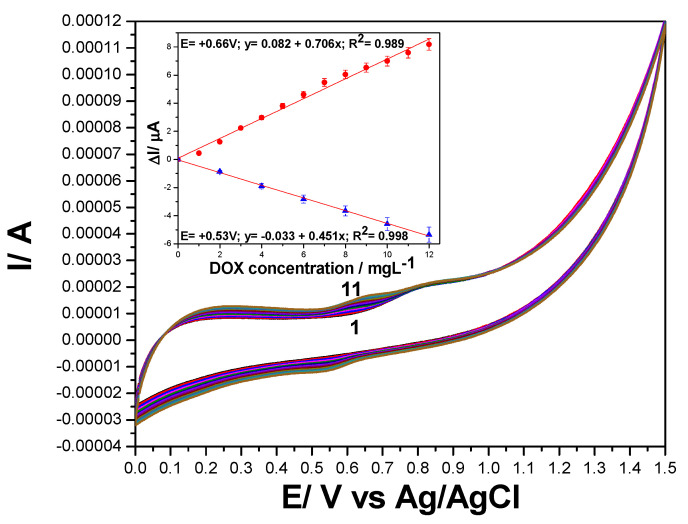
Cyclic voltammograms recorded at GRQD/CNT paste electrode in 0.1 M Na_2_SO_4_ supporting electrolyte (curve 1) and in the presence of various DOX concentrations: 1–10 mg·L^−1^ (curves 2–11), potential scan rate: 0.050 V·s^−1^; potential range: 0.00 to +1.50 V vs. Ag/AgCl. Inset: Calibration plots of current recorded at +0.250 V vs. Ag/AgCl, and +0.660 V vs. Ag/AgCl on anodic branch, and +0.530 V/Ag/AgCl vs. DOX concentration on cathodic branch.

**Figure 5:**
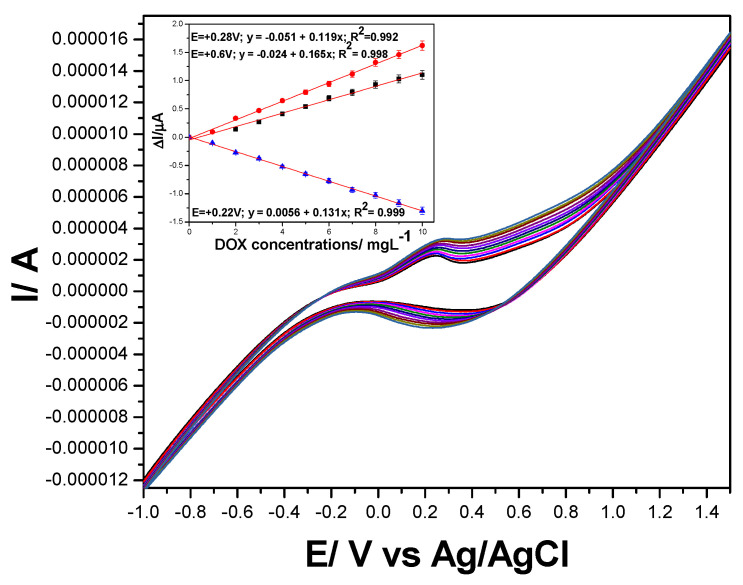
Cyclic voltammograms recorded at Cu/GRQD/CNT paste electrode in 0.1 M NaOH supporting electrolyte (curve 1) and in the presence of various DOX concentrations: 1–10 mg·L^−1^ (curves 2–11), potential scan rate: 0.050 V·s^−1^; potential range from −1.00 to +1.50 V vs. Ag/AgCl. Inset: Calibration plots of current recorded at +0.280 V vs. Ag/AgCl, and +0.600 V vs. Ag/AgCl on anodic branch, and +0.220 V vs. Ag/AgCl vs. DOX concentration on cathodic branch.

The electrochemical behavior of DOX that contains a quinone and a hydroquinone functional groups confers it the possibility to be electrooxidized and/or electroreduced on the electrode material. These functional groups are electroactive on the carbon-based electrodes [11,28,29], and CV shape showed both anodic peak attributed to the DOX electrooxidation and cathodic peak corresponding to the DOX and its oxidation product reduction. This behavior is similar for all carbon-based electrodes tested, whose anodic and cathodic responses depend linearly on the DOX concentration (Figure 4, Figure 5 and Figure S3). Several differences appeared between the electrodes behaviors linked to their compositions, the supporting electrolytes regarding the detection potentials, and the sensitivity, which are gathered in Table 2.

Considering the mechanistic aspects of the DOX electrooxidation and reduction processes, which are responsible for the performance of the electrochemical detection, it can be observed that the rates of the DOX electroreduction processes are lower than the rates of the DOX electrooxidation for the carbon-based electrodes, especially for CNT paste electrode, while similar rates, which are also, responsible for the detection sensitivities were observed for Cu/GRQD/CNT paste electrode. Lower rates of the electroreduction processes on the carbon-based electrodes should be explained by the potential occurrence of other surface-controlled processes or competition with other secondary process as the reduction of DOX oxidation product, which is prior generated during the anodic forward scanning.

Based on the characteristics of the ideal reversible system, ∆E (V) = Ea − Ec = 0.059/n (n-number of electrons), /ia/ic/=1, the reversibility behavior of DOX on Cu/GRQD/CNT is much closer to ideal in comparison with GRQD/CNT and CNT paste electrodes, which should be due to the involvement of Cu(I)/Cu(II) redox couple in DOX electrooxidation and electroreduction. Considering all above presented results related to the detection peculiarities, GRQD/CNT and Cu/GRQD/CNT electrodes are selected for further detection studies.

#### 3.2.1. Mechanistic Aspects—Influence of the Scan Rate

To expound the study of the mechanistic aspects for DOX electrooxidation/electroreduction, the influence of the scan rate on the CV shapes recorded for both GRQD-based electrodes was studied.

**Figure 6:**
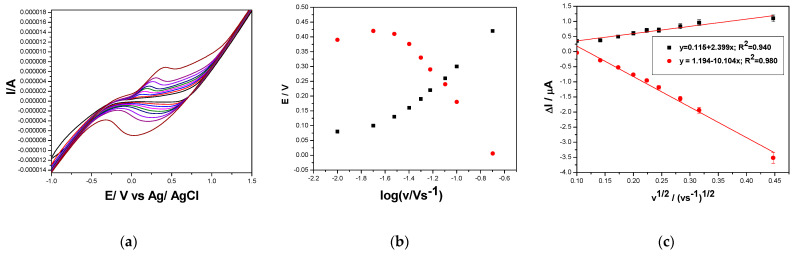
(**a**) Cyclic voltammograms recorded in 5 mg·L^−1^ DOX and 0.1 M NaOH supporting electrolyte with the Cu/GRQD/CNT paste electrode at various scan rates: (curve 1) 10, (curve 2) 20, (curve 3) 30, (curve 4) 40, (curve 5) 50, (curve 6) 60, (curve 7) 80, (curve 8) 100, and (curve 9) 200 mV·s^−1^; (**b**) Dependence of peak potential vs. logarithm of the scan rate. (**c**) Dependence of anodic and cathodic peaks current vs. square root of the scan rate.

Figure 6a–c shows the results of the increasing scan rate ranged from 10 to 200 mV·s^−1^ on CVs recorded on Cu/GRQD/CNT in 0.1 M NaOH and 5 mg·L^−1^ DOX. It can be seen the shifting of the oxidation potential to more positive values and of the reduction potential to more negative values at an increasing scan rate, which shows a deviation from the reversible behavior. This aspect is proved by the anodic and cathodic peak currents (ia and ic), which are differently influenced by the scan rate increasing (Figure 6c). The linear dependence of the anodic and cathodic currents vs. the square root of the scan rate informed about diffusion-controlled processes with the diffusion process rate better for reduction in comparison with anodic one (ia = 2.40 v^1/2^ + 0.115 µA; ic = −10.1 v^1/2^ − (−1.19) µA). For both anodic and cathodic processes, there is no origin interception but in different ways and due to different reasons. For anodic process, positive interception suggests that the surface-controlled aspects cannot be neglected for anodic process, while for cathodic process more complex mechanism is suggested considering the reverse interception.

**Figure 7:**
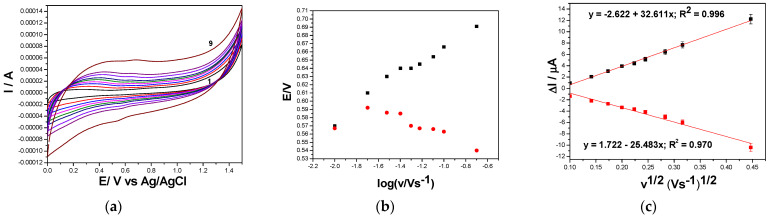
(**a**) Cyclic voltammograms recorded in 5 mg·L^−1^ DOX and 0.1 M Na_2_SO_4_ supporting electrolyte with the GRQD/CNT paste electrode at various scan rates: (curve 1) 10, (curve 2) 20, (curve 3) 30, (curve 4) 40, (curve 5) 50, (curve 6) 60, (curve 7) 80, (curve 8) 100, and (curve 9) 200 mV·s^−1^. (**b**) Dependence of peak potential vs. logarithm of the scan rate. (**c**) Dependence of anodic peak current vs. square root of the scan rate.

The series of CVs recorded on GRQD/CNT paste electrodes at the same scan rate range are presented in Figure 7a. The linear dependences of both anodic and cathodic peaks vs. the square root of the scan rates (Figure 7b) suggest also complex diffusion-controlled processes of DOX electrooxidation and electroreduction on GRQD/CNT electrode (ia = 32.6 v^1/2^ − 2.62 µA; ic = −25.5 v^1/2^ − (−32.6) µA). It must be underlined that in this situation, the diffusion rates of both DOX electrooxidation and electroreduction are almost similar in comparison with Cu/GRQD/CNT electrode.

**Figure 8:**
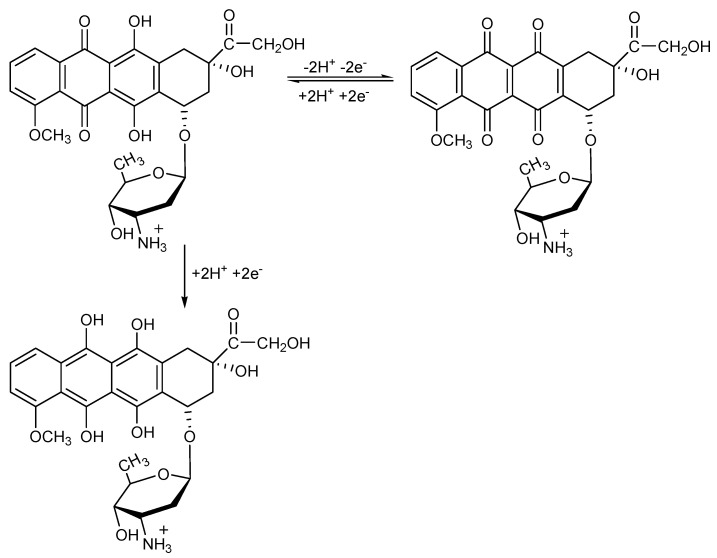
Proposed overall mechanism for the electrochemical oxidation/ reduction of doxorubicin.

Taking into account the mechanisms suggested in the literature considering the reversible two electrons and two protons based DOX electrooxidation/electroreduction [10] and supplementary DOX electroreduction [13], which consider the conversion between quinone and a hydroquinone functional groups, and based on all above presented results, in this study the tentative global mechanism for DOX electrochemical oxidation and reduction is proposed in Figure 8.

The occurrence of two reduction processes is evident for the Cu/GRQD/CNT paste electrode when analyzing the results of the scan rate influence with respect to higher slope for cathodic in comparison with anodic currents.

#### 3.2.2. Optimization of DOX Detection at GRQD Based Paste Electrode

##### Voltammetric Detection of DOX at GRQD-Based Paste Electrodes

Both GRQD/CNT and Cu/GRQD/CNT paste electrodes are further considered for the development of sensitive voltammetric and amperometric detection of DOX.

DPV and SWV are considered advanced voltammetric techniques tested to enhance the sensitivity for DOX detection, taking into consideration their advantages of minimizing the background currents and maximize the faradaic response and implicit, the signal improvement. An amount of 0.1 M Na_2_SO_4_ supporting electrolyte was used for the GRQD/CNT paste electrode and 0.1 M NaOH supporting electrolyte for Cu/GRQD/CNT paste electrode.

To optimize DPV working conditions, modulation amplitudes (MAs) ranged between 2 and 2000 mV and the step potentials (SPs) ranged from 1 to 50 mV were applied. The electrochemical behaviors of the two electrodes were different: stable DPV responses were achieved for GRQD/CNT paste electrode at increasing DOX concentration, and unstable and non-reproducible responses were noticed for Cu/GRQD/CNT paste electrode. Applying DPV technique, a net resulting voltametric current consists of the cumulative effect of both DOX electrooxidation and electroreduction processes expressed in the anodic and cathodic currents, and this type will be determined by the favored process (anodic or cathodic). Probably, for Cu/GRQD/CNT paste electrode, a tight competition between anodic and cathodic processes occurred that confuses each other, leading to the unstable signal.

**Figure 9:**
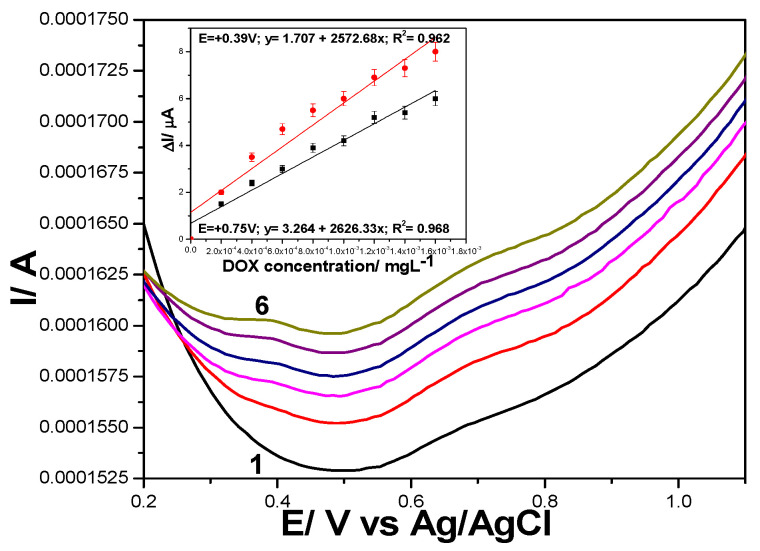
Differential pulse voltammograms recorded at GRQD/CNT paste electrode in 0.1 M Na_2_SO_4_ supporting electrolyte (curve 1) and in the presence of various DOX concentrations: 0.0002–0.0018 mg·L^−1^ (curves 2 to 10) under operating conditions: MA of 0.200 V and SP of 0.010 V; Inset: Calibration plots of current recorded at: (a) +0.390 V vs. Ag/AgCl and (b) + 0.750V vs. Ag/AgCl, vs. DOX concentrations.

Series of DPVs recorded at various DOX concentrations ranged from 0.2 to 1.8 µg·L^−1^ under optimized working conditions of MA of 200 mV and SP of 10 mV for GRQD/CNT paste electrode are presented in Figure 9. It is very interesting that these voltametric conditions allowed to identify the two visible anodic peaks, while only the second was visible by CV (Figure 4). This means that the oxidation of DOX occurs in two stages at +0.390 V vs. Ag/AgCl and respective, at +0.750 V vs. Ag/AgCl. Very high sensitivities were achieved at both oxidation potential values, 2572 µA/mg·L^−1^ at +0.350 V vs. Ag/AgCl and 2626 µA/mg·L^−1^ at +0.750 V vs. Ag/AgCl.

**Figure 10:**
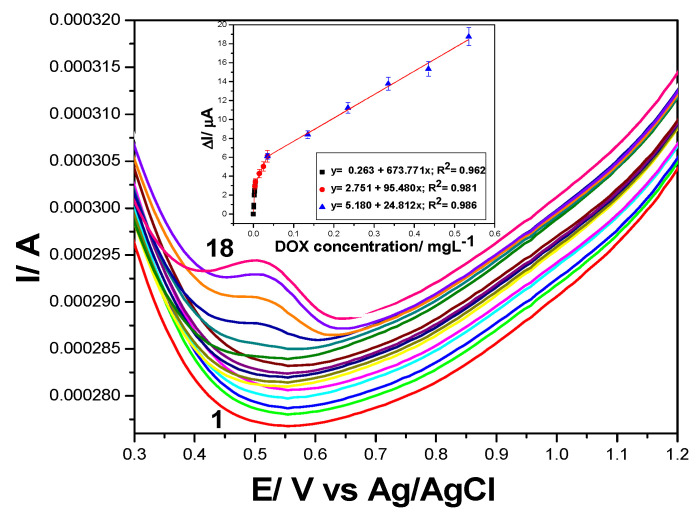
Square wave voltammograms recorded on GRQD/CNT paste electrode in 0.1 M Na_2_SO_4_ supporting electrolyte (curve 1) and in the presence of various DOX concentrations: curves 2–6: 0.0002–0.001 mg·L^−1^ DOX; curves 6–10: 0.001–0.005 mg·L^−1^; curves 10–13: 0.015–0.035 mg·L^−1^; curves 13–18; step potential (SP) 10 mV, modulation amplitude (MA) 200 mV; Hz, and a scan rate of 100 mV·s^−1^, potential range: 0.000 to +1.50 V /Ag/AgCl. Inset: Calibration plots of the currents recorded at E = +0.510 V vs. Ag/ AgCl with DOX concentrations.

Based on the main advantage of SWV to provide fast response, the optimization of working conditions considers the DPV optimum working conditions and the frequency between 2 to 50 Hz. The best results achieved at the frequency of 10 Hz are presented in Figure 10. These working conditions imply the scan rate of 100 mV·s^−1^, at which only the first anodic peak corresponding to the DOX oxidation is visible. This shows that the DOX oxidation rate is lower in the second stage with respect to the first one. Moreover, the detection potential is shifted to more positive value (+0.510 V vs. Ag/AgCl) in comparison to DPV (+0.390 V vs. Ag/AgCl). A large spectrum of DOX concentrations was tested and three concentration ranges resulted after calibration (Inset of Figure 10). The best sensitivity of 674 µA/mg·L^−1^ was achieved for the concentration range from 0.200 to 1.00 µg·L^−1^ DOX, which is about four times lower than 2572 µA/mg·L^−1^ get by DPV.

##### Preconcentration Step Prior to Detection

**Figure 11:**
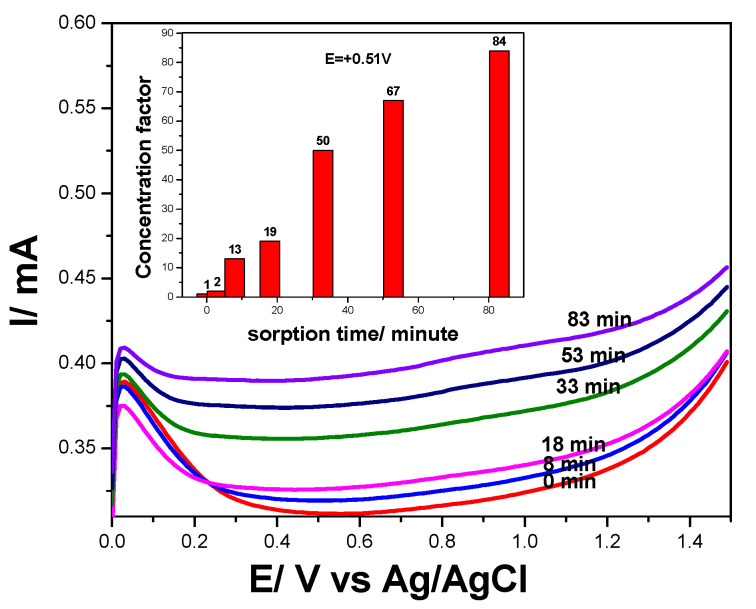
Differential pulse voltammograms recorded on GRQD/CNT paste electrode in the presence of 0.0004 mg·L^−1^ doxorubicin concentrations with different accumulation times; SP of 10 mV, MA of 200 mV; frequency of 10 Hz, and a scan rate of 100 mV·s^−1^, potential range: 0.000 to +1.50 V /Ag/AgCl. Inset: Voltammetric signals achieved by DPV recorded in the presence of 0.0004 mg·L^−1^ DOX in 0.1 M Na_2_SO_4_ supporting electrolyte at GRQD/CNT electrode, as a function of the sorption time in the preconcentration step prior to the detection recorded at E = +0.510 V vs. Ag/AgCl.

Considering the sorption property of the GRQD towards DOX, which should negatively affect the detection of high or quite medium concentration of DOX, the development of a preconcentration-based detection method should allow DOX detection at trace concentration levels. The effect of the sorption time was studied by maintaining the electrode immersed in 0.4 µg·L^−1^ DOX and 0.1 M Na_2_SO_4_ supporting electrolyte at open circuit potential (OCP) for different times. The series of DPVs recorded at different sorption times are presented in Figure 11 and the preconcentration factor is given in Figure 11 inset. A preconcentration factor of about 84 was found after the sorption time of 83 min, which allowed enhancing the sensitivity from 2573 to 216,105 µA/mg·L^−1^.

##### Amperometric Detection of DOX at GRQD-Based Paste Electrodes

For practical applications, it is well-known that the amperometry technique is more convenient due to its simplicity and ease to use. Both GRQD based electrodes were tested with chronoamperometry (CA) and successive additions of specific volumes of DOX in each corresponding supporting electrolyte, 0.1 M Na_2_SO_4_ for GRQD/CNT paste, and 0.1 M NaOH for Cu/GRQD/CNT paste electrodes. No response was found for GRQD/CNT paste electrode using CA technique at all potential levels tested based on CV results as references and quite for higher potential value, at +0.650 V and respective, +0.800 V vs. Ag/AgCl (the results are shown in Supplementary Materials—Figure S4).

**Figure 12:**
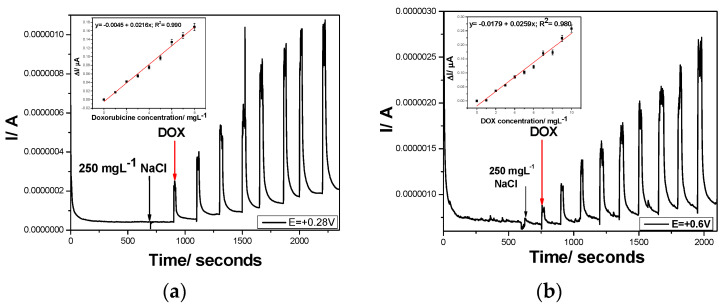
Chronoamperograms (CAs) recorded with the Cu/GRQD/CNT composite electrode in 0.1 M NaOH supporting electrolyte and in the presence of various doxorubicine concentrations: 1–8 mg·L^−1^ for the detection potential level of: (**a**) E = +0.28 V vs. Ag/AgCl. Inset: Calibration plots of current vs. DOX concentration; (**b**) E = +0.6 V vs. Ag/AgCl. Inset: Calibration plots of current vs. DOX concentration.

On the opposite side, Cu/GRQD/CNT paste electrode recorded an increasing current characterized by linear dependence with DOX concentrations by CA at both potential levels selected also based on CV results +0.280 V and at +0.600 V vs. Ag/AgCl, respectively (Figure 12a,b). Similar sensitivities (0.026 µA/mg·L^−1^) were found at the two potential values even if the sensitivities obtained by CV were different (0.119 µA/mg·L^−1^ at +0.280 V vs. Ag/AgCl and 0.165 µA/mg·L^−1^ at +0.600 V vs. Ag/AgCl). It is clearly that the way of DOX electrooxidation/electroreduction process occurrence is different at constant potential in comparison with potential scanning within a potential range, related to the overall current resultant in each situation. Moreover, the electrode fouling occurred by the constant potential maintaining, which is reflected in much lower sensitivity reached for CA in comparison with voltammetric techniques. Considering the potential of chloride interference for real samples of water or human urine, before the first DOX concentration addition, 250 mg·L^−1^ NaCl was added during CA recording and no signal in its presence was founded.

Another amperometric variant to the CA is multiple-pulsed amperometry (MPA), which was proposed considering the two detection potential levels at +0.280 V and +0.600 V vs. Ag/AgCl and more two potential values, one for advanced oxidation of DOX at +1.25 V vs. Ag/AgCl that assured in situ electrode surface cleaning and copper oxides formation and the last one at −0.5 V vs. Ag/AgCl that renews the electrode surface and Cu (I) is reactivated, according with the following scheme:

E1 = −0.500 V vs. Ag/AgCl for 0.2 s—electrode surface renew and Cu (I) maintaining(1)

E2 = +0.280 V vs. Ag/AgCl for 0.15 s—represents the detection potential at which the first stage oxidation of DOX occurred(2)

E3 = +0.600 V vs. Ag/AgCl for 0.15 s—represents the detection potential at which the second stage oxidation of DOX occurred(3)

E4 = +1.250 V vs. Ag/AgCl for 0.15 s—to assure in situ electrode surface cleaning concomitant with copper oxides formation(4)

**Figure 13:**
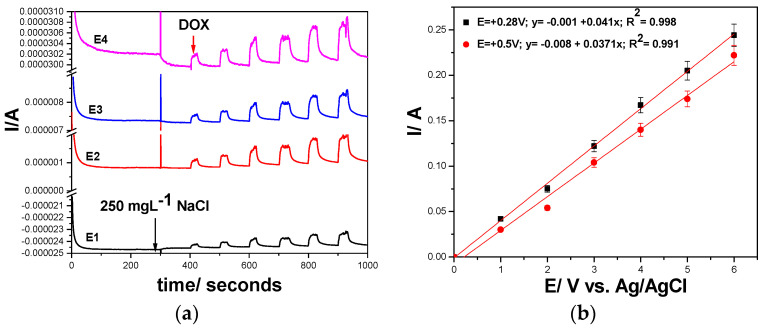
(**a**) Multiple-pulsed amperograms recorded at Cu/GRQD/CNT electrode in 0.1 M NaOH supporting electrolyte and consecutively and continuously adding 1 mg·L^−1^ DOX, recorded at E1 = −0.5 V/Ag/AgCl, E2 = +0.28 V/Ag/AgCl, E3 = +0.6 V/Ag/AgCl, and E4 = +1.25 V/Ag/AgCl in the presence of 250 mg·L^−1^ NaCl; (**b**) Calibration plots of the currents recorded at: E= +0.28 V/ Ag/ AgCl, and E= +0.6 V/Ag/AgCl, versus doxorubicin concentrations.

The amperograms recorded above-presented conditions of the MPA are presented in Figure 13a and the calibration of the linear dependences of the current vs. DOX concentrations at both detection potentials are showed in Figure 13b. In comparison with CA, better sensitivities were reached, which are however, worse than those reached by CV. However, the lowest limit of detection (LOD) is better for amperometric techniques in comparison with CV.

The comparative electroanalytical performances for DOX detection obtained with the two GRQD-based electrodes using all studied electrochemical voltammetric and amperometric techniques in both 0.1 M Na_2_SO_4_ and 0.1 M NaOH supporting electrolytes are gathered in Table 3. The DPV technique achieved the best sensitivity and LOD and LQ at the detection potential value of +0.390 V vs. Ag/AgCl using GRQD/CNT paste electrode. However, for practical application, the accumulation time will be proposed related to the requirements. Good results were obtained by MPA with Cu/GRQD/CNT paste electrode at lower detection potential of +0.280 V vs. Ag/AgCl that mitigate the interference aspects and has the advantage of the method simplicity and lower negative detection potential value.

It is obviously that GRQD/CNT paste electrode allowed the best limit of detection for DOX determination using optimized DPV and preconcentration-DPV methods. In comparison with other carbon-based electrodes reported for DOX detection, GRQD/CNT paste electrodes shows the lowest limit of detection (see Table 4), which makes it to be appropriate for DOX detection at trace levels, as DOX presence has been reported in real surface waters.

All carbon electrodes allowed detecting DOX, but the best sensitivity was reached for GRQD/CNT paste electrode.

In order to test both voltammetric methods using GRQD/CNT paste electrode and amperometric using Cu/GRQD/CNT paste electrode on real samples, 2 mg∙mL^−1^ DOX concentrated solution for infusion was diluted to prepare two ranges of DOX concentrations. One range of DOX solutions of 0.5 µg·L^−1^, 5 µg·L^−1^ and 50 µg·L^−1^ were prepared to test the GRQD/CNT paste electrode with the optimized DPV and the other of 1 mg·L^−1^, 5 mg·L^−1^ and 10 mg·L^−1^ DOX for Cu/GRQD/CNT paste electrode by optimized multiple-pulsed amperometry. The recovery degree was checked by these methods in accordance with the concentration declared by the producer. An average recovery degree of 96% was obtained with GRQD/CNT paste electrode by optimized DPV and 103% for Cu/GRQD/CNT paste by optimized MPA.

Repeatability of the proposed detection procedure was evaluated by comparing the results of the determination of a solution containing 50 µg·L^−1^ DOX with GRQD/CNT paste electrode with DPV and 10 mg·L^−1^ DOX for Cu/GRQD/CNT paste electrode with MPA during the three days. The relative standard deviation less than 5% demonstrated a good repeatability of both proposed GRQD/CNT paste electrode based voltammetric procedure and CuGRQD/CNT paste electrode based amperometric procedure. Finally, the results obtained by these methods for 5 mg·L^−1^ DOX were compared with those obtained by a spectrophotometrical method [45], and it can be concluded that the three methods lead to very close results and that the accuracy of both proposed voltammetric and amperometric methods are good.

## 4. Conclusions

Two types of nanostructured carbon pastes electrodes, graphene quantum dots (GRQD)-carbon nanotubes in paraffin oil (GRQD/CNT) and Cu(I) complex liquid crystalline material-graphene quantum dots (GRQD)-carbon nanotubes in paraffin oil (Cu/GRQD/CNT) electrodes were obtained, characterized morphostructurally, and tested for the electrochemical detection of doxorubicin (DOX)—a commonly used cytostatic in cancer therapy, whose presence was reported in various types of real wastewater and surface waters.

The results of the SEM images and FTIR spectra show both morphological and structural modifications of the Cu/GRQD/CNT paste electrode composition with respect to the individual components, suggesting some kinds of weak intermolecular interactions between Cu complex and functional groups on the carbon surface.

Based on the mechanism elucidation trough cyclic voltammetry (CV), the complex DOX electrooxidation/electroreduction process represents the basis for the development of its voltammetric and amperometric detection with the two GRQD/CNT and Cu/GRQD/CNT paste electrodes. Substitution of a part of GRQD with the Cu(I) complex reduced the active surface area resulting in a lowering of the electrode sensitivity, but permitted a shift of anodic detection potential towards lower values which should avoid the interference of other analytes. The CV results also allowed the selection of the best supporting electrolytes: 0.1 M Na_2_SO_4_ for GRQD/CNT paste electrode and 0.1 M NaOH for Cu/GRQD/CNT paste electrode.

For the detection method development, this study found the DPV and SWV techniques suitable for GRQD/CNT paste electrode to achieve a very nice electroanalytical performance related to the sensitivity and the lowest limit of detection. For this electrode, no stable response was achieved using amperometric techniques. Cu/GRQD/CNT was not able to get a stable voltametric response with the advanced DPV and SWV but by CA and MPA the response was stable. However, the electroanalytical performance was worse in comparison with GRQD/CNT paste electrode, but should allow a better availability for practical detection application methods. Considering the sorption properties of the graphene quantum dots, by applying the preconcentration step before DPV detection, the lowest limit of detection of 1 ng∙L^−1^ was achieved, which is better than the ones reported in the literature for carbon-based electrodes. Both GRQD-based paste electrodes showed great potential for DOX determination in water. The selection of the best electrode composition should rely on the DOX concentration level.

## Figures and Tables

**Table 1:** The weight ratio of the working paste electrodes.

Paste Electrode Type	Weight Ratio, %			
	Paraffin Oil (oil)	Carbon Nanotubes (CNT)	Graphene Quantum Dots (GRQD)	Homoleptic Ionic Cu(I) Coordination Complex Based on 2,2′-Biquinoline Ligand Functionalized with Long Alkyl Chains (Cu)
CNT	66	44	-	-
GRQD/CNT	50	35	15	-
Cu/GRQD/CNT	24.27	6.02	4.24	65

**Table 2:** Detection potential values and the sensitivity for DOX determination.

Electrode Type	Supporting Electrolyte	Anodic		Cathodic		∆E, V vs. Ag/AgCl	$\frac{|\mathrm{ia}|}{|\mathrm{ic}|}$
		E/V vs. Ag/AgCl	Sensitivity/ A/mg·L^−1^	E/V vs. Ag/AgCl	Sensitivity/ A/mg·L^−1^		
Cu/GRQD/CNT	0.1 M NaOH	+0.28	0.12	+0.25	0.13	0.03	0.92
		+0.60	0.16			- **	
GRQD/CNT *	0.1 M Na_2_SO_4_	+0.66	0.71	+0.53	0.46	0.20	1.54
CNT	0.1 M Na_2_SO_4_	+0.35	0.24	+0.22	0.06	0.29	4

* DOX oxidation started at +0.2 V vs. Ag/AgCl but no peak is evidenced, ** no corresponding cathodic peak.

**Table 3:** The electroanalytical parameters for doxorubicin detection with working paste electrode.

Electrode Used	Technique	Potential Detection/V vs. Ag/AgCl	Sensitivity/µA mg·L^−1^	Correlation Coefficient/R^2^	LOD ^(a)^/mg·L^−1^	LQ ^(a)^/mg·L^−1^	RSD ^(b)^ (%)
GRQD/CNT	CV	0.660	0.706	0.989	0.065	0.217	0.194
		0.530 Cathodic branch	0.451	0.998	0.233	0.778	0.507
	DPV	0.390	2572.68	0.962	8.35 × 10^−5^	2.78 × 10^−4^	0.05
	SWV	0.510	673.77	0.962	1.7 × 10^−4^	5.9 × 10^−4^	0.01
	Preconc. DPV	0.390	216,105	0.962	9.94 × 10^−7^	3.31 × 10^−6^	0.05
Cu/GRQD/CNT	CV	0.280	0.119	0.992	3.781	12.605	6.67
		0.600	0.165	0.998	2.758	9.192	5.49
		0.220 Cathodic branch	0.131	0.999	1.172	3.907	4.90
	CA	0.280	0.026	0.996	2.316	7.721	2.99
		0.600	0.026	0.980	0.371	1.236	7.78
	MPA	0.280	0.041	0.998	0.470	1.568	0.79
		0.500	0.037	0.991	3.123	10.411	0.53

(a) LOD- Limit of detection LQ- Limit of quantification (b) RSD- Relative standard deviation determined for three replicates.

**Table 4:** Comparison of performances of GRQD/CNT paste electrode with carbon-based electrodes for electrochemical sensing of doxorubicin.

Method	Electrode	Modifier	LOD (µM)	Matrix	References
CV, DPV	GCE	MWCNT/AgNPs	0.002	-	[40]
DPV	GCE	PS/Fe3O4-GO-SO3H	0.0049	plasma	[15]
DPV	CPE	Carbon paste	0.01	-	[41]
DPV, Impedimetric	GCE	DNA sensors	0.0001	Drugs, artificial plasma	[42]
SWV	HMDE	-	0.1	-	[43]
CV, DPV	GCE	GQD	0.016	plasma	[28]
CV	Pt	MWCNT	0.003	plasma	[44]
DPV	GRQD/CNT	-	1.53 × 10^−4^	water	This work
Preconc. DPW	GRQD/CNT	-	1.83 × 10^−6^	water	This work

## Supplementary Materials

The following are available online at https://www.mdpi.com/article/10.3390/nano11112788/s1, Figure S1: FT-IR spectra of CNT/paraffinic oil (a) and GRQD/CNT/paraffinic oil (b), Figure S2: FT-IR spectra of Cu(I) complex, GRQD/Cu/CNT paste electrode and polarized GRQD/Cu/CNT, Figure S3: Cyclic voltammograms recorded at CNT paste electrode in 0.1 M Na_2_SO_4_ supporting electrolyte (curve 1) and in the present of various doxorubicin concentrations: 1–7 mg·L^−1^ (curves 2–6), potential scan rate: 0.05 V·s^−1^; potential range: −0.5 to +1.5 V/Ag/ AgCl. Inset: Calibration plots of current recorded at +0.34 V/Ag/AgCl on the anodic branch, and +0.47 V/Ag/AgCl on the cathodic branch vs. doxorubicin concentration, Figure S4: Chronoamperograms (CAs) recorded with the GRQD/CNT composite electrode in 0.1 M NaOH supporting electrolyte and in the presence of various doxorubicine concentrations: 1–8 mg·L^−1^ for the detection potential level of: (a) E = +0.8 V vs. Ag/AgCl, and (b) E = +0.65 V vs. Ag/AgCl.
