# Supplementary material for: Graphene Quantum Dots and Cu(I) Liquid Crystal for Advanced Electrochemical Detection of Doxorubicine in Aqueous Solutions
Source: Nanomaterials (Basel). 2021 Oct 21;11(11):2788. doi: 10.3390/nano11112788 (PMC8625772; doi:10.3390/nano11112788)
Supplement: Supplementary file 1 [file nanomaterials-11-02788-s001.zip › nanomaterials-1428789-supplementary.pdf]

nanomaterials

Supplementary Materials

Graphene Quantum Dots and Cu(I) Liquid Crystal for Advanced Electrochemical Detection of Doxorubicine in Aqueous Solutions

Sorina Motoc Ilies [1], Bianca Schinteie [1], Aniela Pop [2], Sorina Negrea [3,4], Carmen Cretu [1], Elisabeta I. Szerb [1,*] and Florica Manea [2,*]

[1] "Coriolan Drăgulescu" Institute of Chemistry, Romanian Academy, 24 Mihai Viteazu Bvd., 300223 Timisoara, Romania; sorinailies@acad-icht.tm.edu.ro (S.M.I.); biancab@acad-icht.tm.edu.ro (B.S.); cretucarmen@acad-icht.tm.edu.ro (C.C.)

[2] Department of Applied Chemistry and Engineering of Inorganic Compounds and Environment, Politehnica University of Timisoara, 2 Victoriei Square, 300006 Timisoara, Romania; aniela.pop@upt.ro

[3] National Institute of Research and Development for Industrial Ecology (INCD ECOIND), Timisoara Branch, 300431 Timisoara, Romania; negrea.sorina@yahoo.com

[4] Department of Environmental Engineering and Management, "Gheorghe Asachi" Technical University of Iasi, 700050 Iasi, Romania

* Correspondence: eszerb@acad-icht.tm.edu.ro (E.I.S.); florica.manea@upt.ro (F.M.)

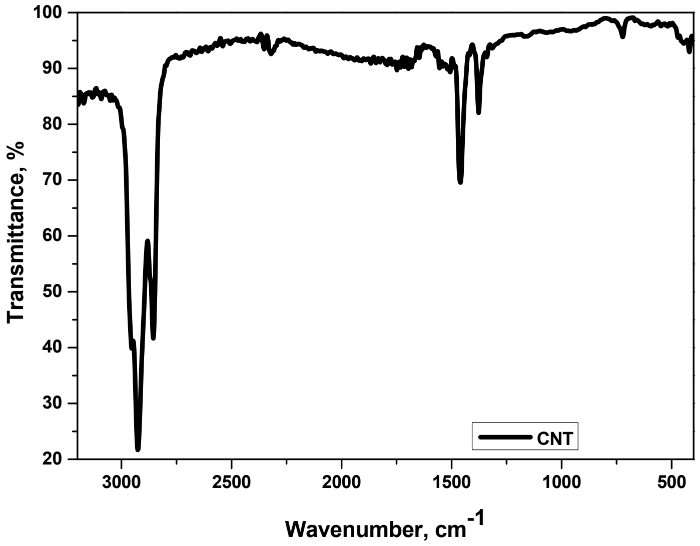

(**a**)

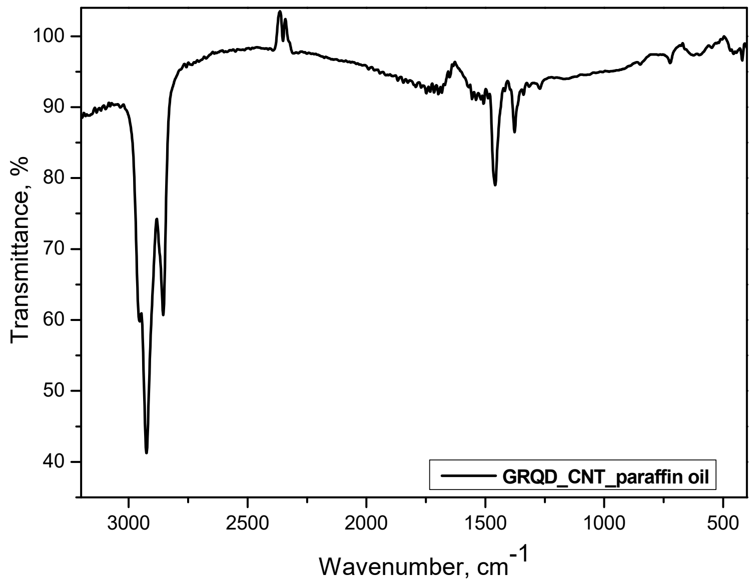

(**b**)

Figure S1. FT-IR spectra of CNT/paraffinic oil (**a**) and GRQD/CNT/paraffinic oil (**b**).

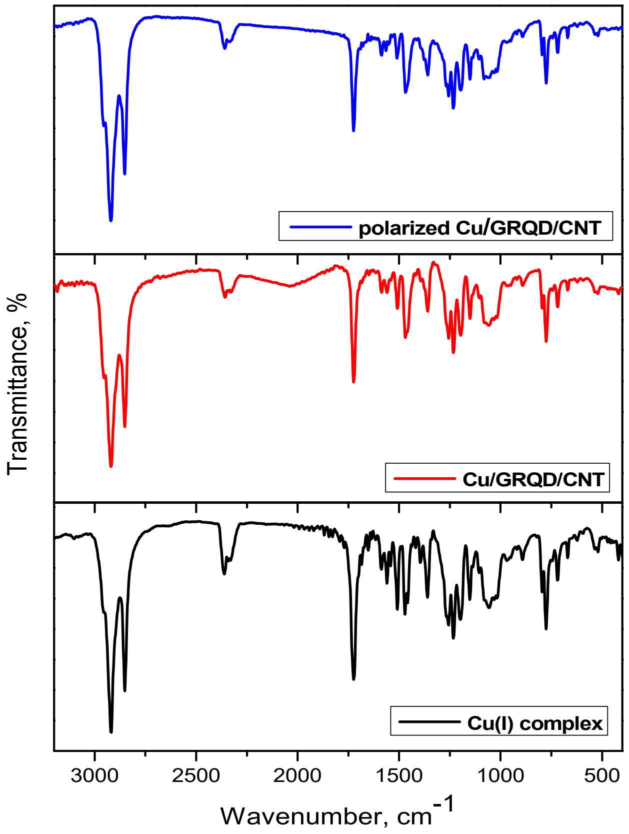

Figure S2. FT-IR spectra of Cu(I) complex, GRQD/Cu/CNT paste electrode and polarized GRQD/Cu/CNT.

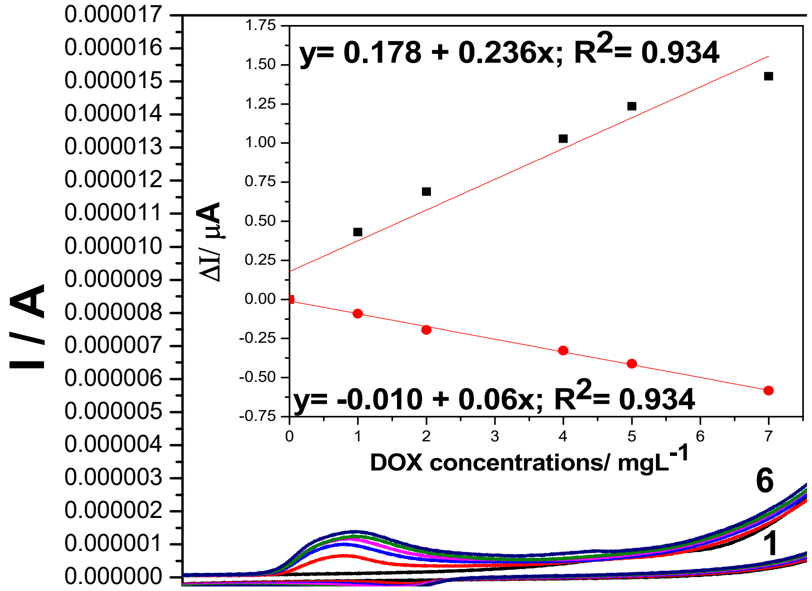

Figure S3. Cyclic voltammograms recorded at CNT paste electrode in 0.1 M Na_2SO_4 supporting electrolyte (curve 1) and in the present of various doxorubicin concentrations: 1–7 mgL^{-1} (curves 2–6), potential scan rate: 0.05 $V{*}s^{-1}$; potential range: −0.5 to +1.5 V/Ag/ AgCl. Inset: Calibration plots of current recorded at +0.34 V/Ag/AgCl on the anodic branch, and +0.47 V/Ag/AgCl on the cathodic branch vs. doxorubicin concentration.

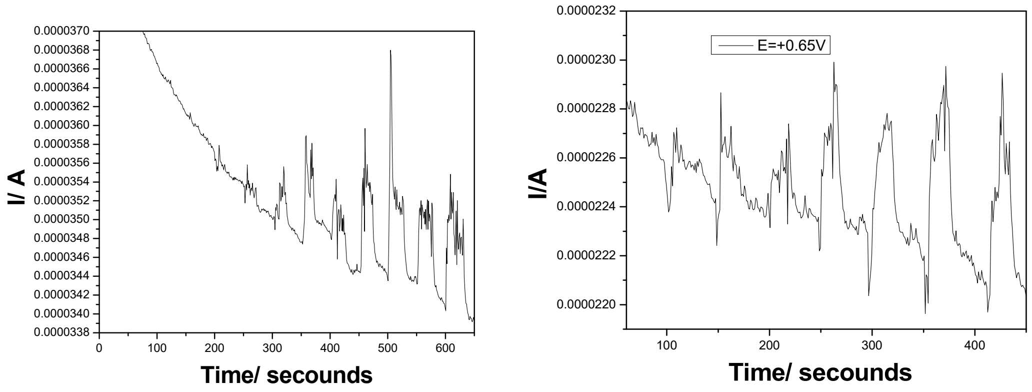

(**a**) (**b**)

Figure S4. Chronoamperograms (CAs) recorded with the GRQD/CNT composite electrode in 0.1 M NaOH supporting electrolyte and in the presence of various doxorubicine concentrations: 1–8 $mgL^{-1}$for the detection potential level of: (a) E= +0.8 V vs. Ag/AgCl, and (b) E= +0.65 V vs. Ag/AgCl.
